# Serum lipid alterations identified in chronic hepatitis B, hepatitis B virus-associated cirrhosis and carcinoma patients

**DOI:** 10.1038/srep42710

**Published:** 2017-02-15

**Authors:** Tao Wu, Xiaojiao Zheng, Ming Yang, Aihua Zhao, Meng Li, Tianlu Chen, Jun Panee, Wei Jia, Guang Ji

**Affiliations:** 1 Center of Chinese Medical Therapy and Systems Biology, Shanghai University of Traditional Chinese Medicine, Shanghai 201203, China; 2 Institute of Digestive Disease, Longhua Hospital, Shanghai University of Traditional Chinese Medicine, Shanghai 200032, China; 3 Shanghai Key Laboratory of Diabetes Mellitus and Center for Translational Medicine, Shanghai Jiao Tong University Affiliated Sixth People’s Hospital, Shanghai 200233, China; 4 Department of Cell and Molecular Biology, John A. Burns School of Medicine, University of Hawaii at Monoa, Honolulu, Hawaii 96813, United States; 5 University of Hawaii Cancer Center, Honolulu, Hawaii 96813, United States; 6 E-institute of Shanghai Municipal Education Committee, Shanghai University of Traditional Chinese Medicine, Shanghai 201203, China

## Abstract

The incidences of chronic hepatitis B (CHB), Hepatitis B virus (HBV)-associated cirrhosis and HBV-associated carcinoma are high and increasing. This study was designed to evaluate serum lipid metabolite changes that are associated with the progression from CHB to HBV-associated cirrhosis and ultimately to HBV-associated HCC. A targeted metabolomic assay was performed in fasting sera from 136 CHB patients, 104 HBV-associated cirrhosis, and 95 HBV-associated HCC using ultra-performance liquid chromatography triple quadrupole mass spectrometry. A total of 140 metabolites were identified. Clear separations between each two groups were obtained using the partial least squares discriminate analysis of 9 lipid metabolites. Progressively lower levels of long-chain lysophosphatidylcholines (lysoPC a C18:2, lysoPC a C20:3, lysoPC a C20:4) were observed from CHB to cirrhosis to carcinoma; lower levels of lysoPC a C20:4 were found in patients with higher model for end-stage liver disease in the same disease group; and lysoPC a C20:3 levels were lower in Child-Pugh Class C than in Class A and Class B in HBV-associated cirrhosis and HBV-associated HCC groups. The octadecadienyl carnitine level was higher in HBV-associated cirrhosis group than in other two groups. Serum levels of selected long-chain lysoPCs are promising markers for the progression of HBV-associated liver diseases.

Approximately 500 million individuals are chronically infected with hepatitis B virus (HBV) or hepatitis C virus (HCV) worldwide, and almost 1 million people die from causes related to chronic viral hepatitis each year^1^. Although an effective HBV vaccine has been developed, it has not been made available to everyone and there are many HBV carriers who are still at increased risk of developing cirrhosis^2^. Liver cirrhosis is responsible for 80% of hepatocarcinoma (HCC) incidence^3^.

The prevalence of HCC is increasing worldwide^4^. When HCC is diagnosed at an early stage, resection or liver transplantation can be an effective treatment. However, the diagnosis of HCC often occurs when surgery is no longer an option^5^. Therefore, there is an increasing focus on the development of non-invasive techniques and identification of early biomarkers for the diagnosis and treatment of HBV-associated cirrhosis and carcinoma.

Cellular metabolites constantly undergo flux and many of them can be detected in serum or other body fluids. Thus they can be utilized as sensitive markers of patient metabolic status^6^,^7^. Metabolomics is a growing high-throughput technology used to study systemic metabolism^8^, and has been applied in disease differentiation, clustering different subgroups of a disease, drug development or drug-response, and drug toxicity^9^. Targeted metabolomics simultaneously measures a large number of metabolites, can identify and quantify metabolites related to specific disease conditions^10^, and has been successfully used in several studies^11^,^12^. One of the analytical platforms of metabolomics, liquid chromatography-mass spectrometry (LC-MS), has been extensively used to identify early diagnosis biomarkers of HCC in serum, plasma, urine and fecal samples^13^,^14^,^15^,^16^,^17^,^18^,^19^,^20^.

The metabolite markers that have been previously identified in HCC are involved in key metabolic pathways, such as the metabolism of bile acid, phospholipids (PL), fatty acids (FA), and methionine, as well as glycolysis and urea cycle^3^. Previous studies have shown decreased serum levels of lysoPCs in non-alcoholic fatty liver disease, chronic hepatitis B(CHB), cirrhosis and HCC relative to healthy controls^17^,^21^,^22^,^23^. What is still lacking, however, is a biomarker that clearly distinguishes CHB, HBV-associated cirrhosis and HBV-associated HCC from one another.

Our hypothesis for this investigation was that metabolic alterations of lipids, particularly those involved in hepatocyte membrane structure and secretion of lipids into the systemic circulation, may precede the development of HBV-induced hepatitis, cirrhosis and carcinoma. Furthermore, the disturbed lipid metabolism would be reflected by changes in serum lipid metabolite concentrations, notably levels of lysoPCs, phosphatidylcholines (PCs), acylcarnitines (AC), and sphingomyelins (SM). To test this hypothesis, we used a targeted metabolomics approach. An ultra-performance liquid chromatography triple quadrupole mass spectrometry (UPLC-TQMS) platform was employed to measure serum samples collected from patients with CHB, HBV-associated cirrhosis and HBV-associated HCC, respectively.

The aim of this study was to discover novel metabolite markers which reflect the dynamic metabolic changes during the progression from CHB to HBV-associated cirrhosis to HBV-associated HCC, and to obtain insights into the possible molecular mechanism responsible for these changes for a given stage of hepatitis B associated liver disease.

## Results

### Demographic information and clinical characteristics of patients

Demographic, clinical and serological data of subjects in the three groups are summarized in Table 1. The 335 patients were separated into 3 groups: CHB group (n = 136), HBV-associated cirrhosis group (n = 104) and HBV-associated HCC group (n = 95). All patients were positive for hepatitis B surface antigen (HBsAg).

The mean age of the HBV-associated cirrhosis group (56.5 y) and HBV-associated HCC group (56 y) were older than that of CHB group (41 y). Male subjects accounted for 85.2% in HBV-associated HCC group, which was higher than those in HBV-associated cirrhosis group (58.6%) and CHB group (62.5%). The body mass index (BMI) of the HBV-associated HCC group (22.3 kg/m^2^) was lower than those of HBV-associated cirrhosis group (23.4 kg/m^2^) and CHB group (23.7 kg/m^2^). Serum levels of total bilirubin (TBIL), alkaline phosphatase (ALP), total bile acid (TBA), prothrombin time (PT), international normalized ratio (INR), carcino embryonie antigen (CEA) and model for end-stage liver disease (MELD) Score were lower in CHB group than HBV-associated cirrhosis and HBV-associated HCC groups, while levels of total protein (TP), albumin (ALB), lactate dehydrogenase (LDH), choline esterase (CHE), cholesterol (TC), triglyceride (TG), red blood cell (RBC), platelet (PLT) were higher for CHB relative to HBV-associated cirrhosis and HBV-associated HCC groups. The cirrhosis group had lower alanine transaminase (ALT) level than the CHB group. Serum levels of direct bilirubin (DBIL), gamma-glutamyl transferase (GGT), creatinine (CREA) and alpha-fetoprotein (AFP) in the HBV-associated HCC group were higher than those in the CHB group. Serum levels of ALP, GGT, TP, TC, AFP, hemoglobin (HGB), PLT were lower, but the MELD Score class and stage were higher in HBV-associated cirrhosis group than those in HBV-associated HCC group. The proportion of positive HBV desoxyribonucleic acid (HBV-DNA) was higher in CHB group than that in either the HBV-associated cirrhosis or HBV-associated HCC groups. No significant differences were observed for aspartate transaminase (AST) and glucose (GLU) among all 3 groups.

### Serum lipid profiles varied among CHB, LC and HCC groups

**Figure 1:**
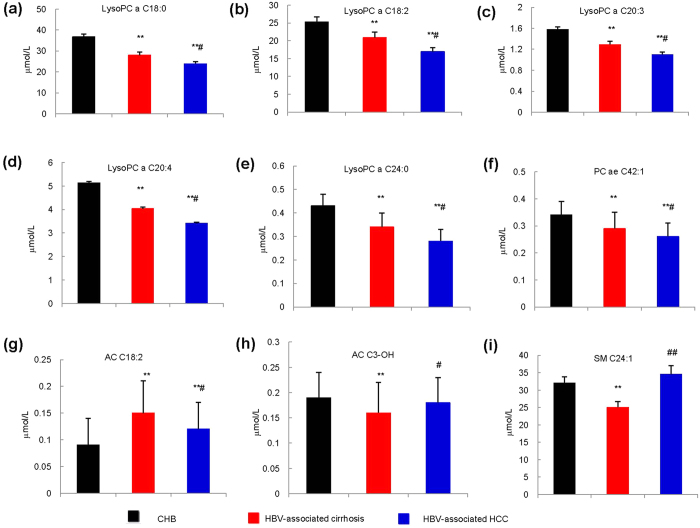
Observed changes of 9 metabolites in the sera of CHB, HBV-associated cirrhosis and HBV-associated HCC patients (N = 136, 104, 95 respectively). Serum concentrations of lipid metabolites were detected using UPLC-TQMS. (**a–f**) LysoPC and PC: lysoPC a C18:0, lysoPC a C18:2, lysoPC a C20:3, lysoPC a C20:4, lysoPC a C24:0, PC ae C42:1 respectively. (**g,h**) AC: AC C18:2, AC C3-OH. (**i**) SM: SM C24:1. *P* values were calculated from non-parametric Kruskal-Wallis test and adjusted by the FDR method. *p < 0.05; **p < 0.01; ***p < 0.001 when compared to CHB. ^#^*p* < 0.05; ^##^*p* < 0.01; ^###^*p* < 0.001 when compared to HBV-associated cirrhosis.

**Figure 2:**
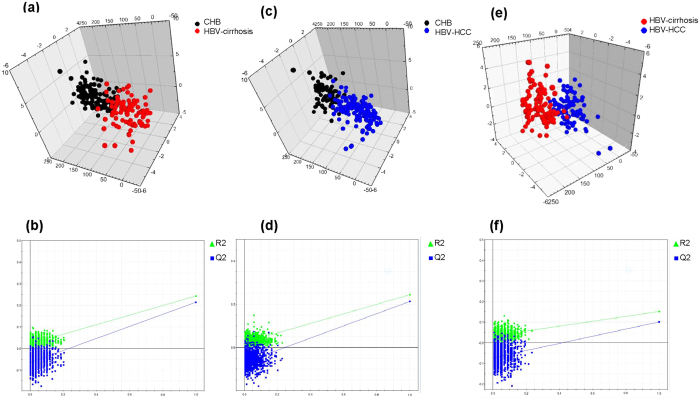
3D PLS-DA Score plots show clear separation within each two groups based on the selected 9 representative variables. CHB versus HBV-associated cirrhosis: (**a**) PLS-DA scores plot: R2X = 0.564 R2Y = 0.243 Q2 = 0.214. (**b**) Permutation analysis. CHB versus HBV-associated HCC: (**c**) PLS-DA scores plot: R2X = 0.569 R2Y = 0.244 Q2 = 0.212. (**d**) Permutation analysis. HBV-associated cirrhosis versus HBV-associated HCC: (**e**) PLS-DA scores plot: R2X = 0.548 R2Y = 0.152 Q2 = 0.101. (**f**) Permutation analysis.

The Z-score plot shows the variation of 140 metabolites in patients with CHB, HBV-associated cirrhosis, and HBV-associated HCC (Supplementary Table 1 and Supplementary Fig. S1A). Principal component analysis (PCA) and partial least squares discriminant analysis (PLS-DA) scores plots among groups and within each two groups are shown in Supplementary Fig. S1B–D and Supplementary Fig. S2A–J. Table 2 shows that serum levels of five lysoPC species (lysoPC a C18:0, lysoPC a C18:2, lysoPC a C20:3, lysoPC a C20:4, lysoPC a C24:0) and one PC (PC ae C42:1) decreased gradually from CHB to HBV-associated cirrhosis and to HBV-associated HCC. Serum levels of AC C18:2 was higher but those of AC C3-OH and SM C24:1 were lower in the HBV-associated cirrhosis group than in either CHB or HBV-associated HCC groups. The group differences of the 9 metabolites are further visualized in Table 2 and Fig. 1a–i. PLS-DA scores plots show clear separation within each two groups based on 9 metabolites: CHB versus HBV-associated cirrhosis (Fig. 2a,b, R2X = 0.564 R2Y = 0.243 Q2 = 0.214), CHB versus HBV-associated HCC (Fig. 2c,d, R2X = 0.569 R2Y = 0.244 Q2 = 0.212), HBV-associated cirrhosis versus HBV-associated HCC (Fig. 2e,f, R2X = 0.548 R2Y = 0.152 Q2 = 0.101), suggesting that these metabolites are promising markers for disease progression. To visualize the extent of the observed changes, a heat map was used to illustrate the fold of change (FC) of the 9 metabolites among the three groups with CHB as baseline (Supplementary Fig. S3). The results of multiple logistic regression assessing the association between the 9 lipids and diseases, adjusted for age, gender and BMI are summarized in Supplementary Table 2. Four metabolites including lysoPC a C18:2, lysoPC a C20:3, lysoPC a C20:4 and AC C18:2 were found to associate with all three groups (Table 2).

### LysoPC a C20:3 varied with Child-Pugh Class in patients with HBV-associated cirrhosis and HBV-associated HCC

Child-Pugh score is a commonly used model to assess the severity of liver disease. We further analyzed the association between the 9 metabolites and the Child-Pugh Class that includes A (5–6), B (7–9) and C (10–15). Among the 9 metabolites, lysoPC a C20:3 was found significantly lower in Class C than in Class A and Class B in both HBV-associated cirrhosis and HBV-associated HCC groups (Table 3). A similar trend was observed for lysoPC a C18:0, C18:2, C20:4 and C24:0 but the decrease in their respective values did not quite reach statistical significance. We further performed Spearman correlation analysis between lysoPC a C20:3 and Child-Pugh scores, as well as ALT, AST, TBIL, DBIL, ALP, GGT, TP, ALB, LDH, TBA, CHE, TC, TG, CREA, GLU, PT, INR, AFP, CEA, Viral load, RBC, HGB, and PLT. The detailed correlation coefficient data and *p*-values between lysoPC a C20:3 and other variables both in HBV-associated cirrhosis and HBV-associated HCC were provided in Supplementary Table 3. Serum lysoPC a C20:3 was found positively correlated with the levels of ALB, CHE, RBC, HGB, and negatively correlated with Child-Pugh scores, TBIL, DBIL, LDH, PT, and INR. The result suggests that serum lysoPC a C20:3 level could reflect the metabolic function of liver and hepatocellular injury, which further supports lysoPC a C20:3 as a potential biomarker for HBV-associated disease progression.

### Changes of lysoPC a C20:4 was associated with MELD Score and disease progression

MELD score is a commonly used clinical indicator that reflects the severity of liver injury. We performed a sub-class analysis in patients with CHB, HBV-associated cirrhosis and HBV-associated HCC according to their MELD scores (≤8.99 or >8.99) to evaluate the correlation between MELD score and serum lipid levels. We found that serum lysoPC a C20:4 decreased with increased MELD score and with the disease progression from CHB to HBV-associated cirrhosis, and to HBV-associated HCC (Table 4).

### Changes of lysoPC a C18:2, lysoPC a C20:3, lysoPC a C20:4 were associated with the progression in HBVDNA positive and negative groups

It is well known that HBsAg particles are partially composed of lipids, so we further analyzed its influence on serum levels of the selected metabolites. The HBV-DNA content of >=1E + 03 was considered as HBV-DNA positive^9^. We classified patients into two subgroups according to their HBVDNA levels, ie HBVDNA negative group (<1E + 03) and HBVDNA positive group (>=1E + 03) and then analyzed the serum concentrations of the selected 9 metabolites. Significant differences of the selected metabolites were found among the comparisons of CHB, HBV-associated cirrhosis and HBV-associated HCC either in the HBVDNA negative or in the HBVDNA positive groups (Table 5).

## Discussion

Lipids are not only components of cell membranes but are also involved in signal transduction^24^. PCs, lysoPCs, and SMs are the three major phospholipid groups that are important components of serum lipoproteins and cell membranes. They are involved in regulation of cell functions, membrane protein trafficking and inflammation^25^. In human plasma, PCs, lysoPCs, and SMs comprise approximately 60–70%, 10–20%, and 10–20% of circulating PLs, respectively. In HBV-related diseases, PCs account for more than 80% of total membrane lipids of the HBsAg^26^.

**Figure 3:**
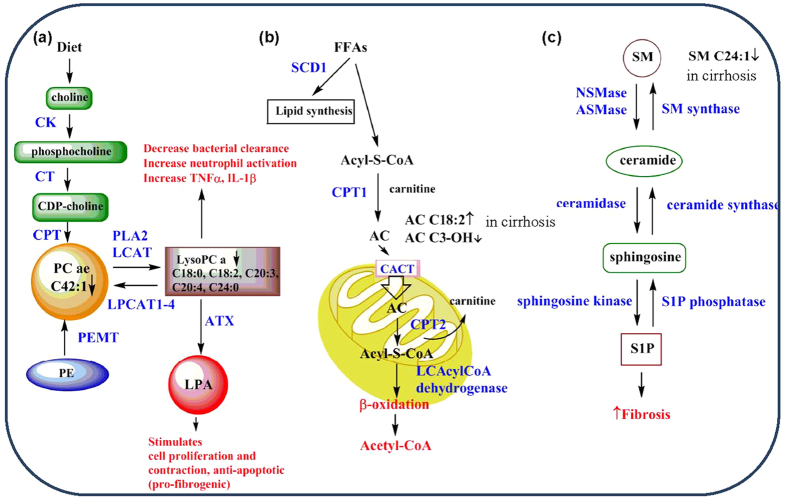
Diagrammatic drawing of possible altered serum lipid metabolic pathways in CHB, HBV-associated cirrhosis and HBV-associated HCC. Blue type = enzyme. (**a**) The 3 dominant pathways leading to the production of PC. LysoPCs can be used to make PC or LPA. The effect of decreased lysoPC and production of LPA are typed in red. (**b**) The carnitine shuttle is used to transport long chain FAs into the mitochondria to undergo β-oxidation which ultimately leads to the final oxidation product (in red), acetyl-CoA. (**c**) Pathway for the metabolism of SMs (and vice versa) to S1P, an important regulator in the liver for fibrosis. CK, choline kinase; CT, Cytidine; CDP-choline, cytidine-5′-diphosphocholine; CPT, CDP-choline:1,2-diacylglycerol cholinephosphotransferase; PEMT, phosphatidylethanolamine N-methyltransferase; PLA2, phospholipase-α2; LCAT, lecithin-cholesterol acyltransferase; LPCAT, lysophosphatidylcholine acyltransferase; LPA, lysophosphatidic acid; PC, phospatidylcholine; PE, phosphotidylethanolamine; LysoPC, lysophosphatidylcholine; TNF-α, tumor necrosis factor-α; IL-1β, interleukin-1β; SCD1, stearoyl-CoA desaturase 1; CPT1,2, carnitine palmitoyltransferase 1,2; CACT, carnitine-acylcarnitine translocase; LCAcylCoA dehydrogenase, long chain acyl-CoA dehydrogenase; NSMase, neutral sphingomyelinase; ASMase, acidic sphingomyelinase; SM synthase, sphingomyelin synthase; S1P, sphingosine-1-phosphate.

In the present study, we observed significant alterations in the HBV-associated cirrhosis and HBV-associated HCC groups relative to CHB. LysoPC 13/14(93%), PC 56/73(77%), AC 8/36(22%) and SM 10/12(83%) suggesting a general disturbance in the synthesis, transport and/or breakdown of lipids in patients as liver disease progresses from CHB to HBV-associated cirrhosis and/or HBV-associated HCC (Supplementary Table 1, Supplementary Fig. S1 and Supplementary Fig. S2). Nine out of 140 measured metabolites were significantly different between CHB and HBV-associated cirrhosis, and between HBV-associated cirrhosis and HBV-associated HCC (Table 2, Figs 1 and 2). Among the 9 metabolites, serum levels of five types of lysoPC (lysoPC a C18:0, lysoPC a C18:2, lysoPC a C20:3, lysoPC a C20:4, lysoPC a C24:0), and one PC (PC ae C42:1) were found to decrease gradually from CHB to HBV-associated cirrhosis to HBV-associated HCC. LysoPC a C20:3 was found significantly lower in Class C than in Class A and Class B in both HBV-associated cirrhosis and HBV-associated HCC groups (Table 3), lysoPC a C20:4 decreased with increased MELD score in 3 groups (Table 4). The selected metabolites were still significantly different between groups under the circumstance of HBVDNA negative or HBVDNA positive which indicated the metabolic changes did not affected by HBV (Table 5). An illustration of the possible perturbed lipid metabolism pathways and their influence on liver disease progression is shown in Fig. 3.

The first pathway described in Fig. 3a is the PE/cytidine-5′-diphosphocholine (CDP-choline) → PC → LysoPC → lysophosphatidic acid (LPA). The PE/phosphatidylethanolamine N-methyltransferase (PEMT) → PC pathway accounts for about 30% of PC production has been shown to be decreased in activity in early HCC and disappeared in later stages^27^,^28^. Loss of the PE → PC pathway has also been associated with increased activity of the CDP-choline pathway and increased hepatocyte cell proliferation whereas PEMT expression has been shown to inhibit the CDP-choline pathway and acts as a negative regulator of hepatocyte cell proliferation, a potential tumor suppressive action^29^. In this study, 77% of the PC compounds measured for HCC and LC (both highly cell proliferative states), showed significant decreases in their serum concentrations relative to CHB perhaps indicating some loss of the PE → PC pathway due to decreased PEMT expression or via possible retention of PCs in the liver tissue. Studies of PEMT^−/−^ mice indicated that PEMT deficiency reduces the PC/PE ratio which had the effect of making these mice more susceptible to endoplasmic reticulum stress and steatohepatitis^28^. Additionally, a previous study using imaging mass spectrometry revealed enrichment of certain PC species and reduction of others in HCC tissue which may also explain low serum concentrations of PCs^30^. Further studies on the hepatic expression of PEMT as well as tissue levels of PCs with respect to PC serum concentrations are warranted for future investigation. In this study, a low serum level of a specific PC, PC ae 42:1, was found to be significantly different between CHB vs. HBV-associated cirrhosis, HBV-associated HCC and HBV-associated cirrhosis vs. HBV-associated HCC meaning that this serum PC level may prove useful as a biomarker in staging liver disease progression.

Approximately 93% of the measured serum lysoPCs (13/14) were also significantly decreased for HBV-associated cirrhosis and HBV-associated HCC relative to CHB and several possibilities exist for this general decrease to happen (Fig. 3a). Factors that may be involved include: decreased PC availability due to loss of PEMT activity, decreased expression of phospholipase-α2 (PLA2) and/or lecithin-cholesterol acyltransferase (LCAT), increased expression of lysophosphatidylcholine acyltransferase 1–4 (LPCAT1–4) or increased expression of autotaxin (ATX) (Fig. 3). LPCAT1 overexpression in Huh7 and HepG2 cells has been shown to increase cell proliferation and invasion by enriching certain cell membrane PCs^30^. This same PC enrichment was observed in human HCC tissue samples via imaging mass spectrometry^30^. ATX has been documented to be highly expressed in a variety of malignancies including HCC and has been considered a predictive biomarker for liver cirrhosis^31^,^32^. Further research addressing correlations between hepatic tissue expression of enzymes in the PC ↔ lysoPC → LPA and serum levels with respect to CHB and CHB induced LC and HCC will need to be done to determine the reason for generally decreased serum lysoPCs observed in this study. Five lysoPCs were found to be significantly decreased in CHB vs. HBV-associated cirrhosis, HBV-associated HCC and HBV-associated cirrhosis vs. HBV-associated HCC revealing 5 potential biomarkers for Hepatitis B induced liver disease progression.

Our overall observations are consistent with several studies reporting decreased PCs and lysoPCs in the circulation during HBV-induced liver disease progression^17^. Serum levels of four lysoPCs were decreased in patients with HCC and HBV-associated cirrhosis compared with healthy controls^13^. Two of these lysoPCs (lysoPC a 18:0 and lysoPC a 18:2) were found decreased for increasing stages of liver disease. Previously, in two additional studies, decreased serum lysoPCs and increased serum bile acids were detected in patients with HBV-associated cirrhosis^33^ as well as, lower serum PCs in patients with cirrhosis^34^ relative to healthy controls. Yang *et al*. suggested that decreased serum levels of lysoPC a C16:0, lysoPC a C18:0, lysoPC a C18:1 and lysoPC a C18:2, together with increased serum levels of glycoehenodeoxycholie acid (GCDCA) or its isomer glycodeoxycholic acid (GDCA) were potential biomarkers for acute deterioration of liver function in CHB^33^. However, other studies have documented that some serum lysoPCs increased with progressions of HBV-induced liver diseases, including lysoPC a C18:0^35^, taurocholic acid, lysophosphoethanolamine C16:0, and lysoPC a C22:5^18^, sphingosine-1-phosphate (S-1-P) and lysoPC C17:0^3^. The great difference between our study and these previous studies is that we have focused on hepatitis, cirrhosis and carcinoma patients that are all triggered by HBV, and to the best of our knowledge, this is the first study that systematically evaluated serum lipid levels including not only lysoPCs but also SMs and ACs from patients in different stages of liver diseases.

LysoPC has stimulatory effects on immune cells, including monocytes, macrophages and neutrophils^33^. It can increase bacterial clearance and reduce neutrophil deactivation, tumour necrosis factor-α (TNFα) and interleukin-1β (IL-1β) levels^36^. LysoPC is also a major source of LPA, which stimulates cell proliferation, and aberrant LPA-signaling has been linked to cancer^37^. Choline kinase alpha (CK) is an enzyme catalyzing the first step of PC biosynthesis (Fig. 3a), and silencing CK expression was shown to inhibit HBV replication, indicating that PC may affect the replication of HBV^38^. Furthermore, altered biogenesis of PC may also contribute to defects in regeneration processes after HBV-induced liver injury^39^. These studies have provided evidence that PC or lysoPC dysregulation may contribute to the progression of CHB to cirrhosis and HCC through multiple pathways.

Carnitine and ACs are involved in FA oxidation and organic acid metabolism, and are considered important biomarkers for metabolic disorders such as obesity, diabetes, hepatic encephalopathy and cardiomyopathy^40^,^41^. Long-chain FAs are unable to cross the inner mitochondrial membrane without esterification in the cytosole (Fig. 3b) in an ATP-consuming step to Acyl-S-CoA followed by biotransformation to ACs by means of carnitine palmitoyltransferase 1 (CPT1)^42^. ACs are transported into the mitochondria by facilitated diffusion via the enzyme, carnitine/AC translocase (CACT)^43^. Once inside the mitochondrial matrix, the enzyme CPT2 reforms Acyl-S-CoA and carnitine is shuttled back out of the matrix via CACT. In this study 22% of the ACs were found to be significantly altered between CHB and later stages of liver disease, HBV-associated cirrhosis and HBV-associated HCC. Carnitine, AC C0, 2 short chain ACs, and 4 medium chain ACs were found to be significantly decreased for both LC and HCC relative to CHB patients. Our study showed that serum concentrations of AC C18:2 were higher and that levels of AC C3-OH were lower in LC patients than in both CHB and HCC patients (Table 2, Fig. 1; Supplementary Fig. S3). Elevated serum levels of long chain ACs and decreased levels of medium and short chain ACs have been previously reported for cirrhosis and HCC^44^.

The activities of several enzymes involved in AC metabolism (Fig. 3b) can be estimated by ratios, such as (AC C16 + AC C18)/carnitine for CPT1, (AC C16 + AC C18:1)/AC C2 for CPT2, AC C16/AC C8 for long chain AcylCoA dehydrogenase, AC C2/carnitine for β-oxidation of even-numbered FAs (Supplementary Table 4). The changes of (AC C16 + AC C18)/carnitine hint that activity of CPT1 had a trend of increase with disease progression. The ratio of (AC C16 + AC C18:1)/AC C2 in HBV-associated cirrhosis was higher than those in CHB and HBV-associated HCC, which indicates an increase for the cirrhosis stage of disease progression for CPT2 activity. This particular increase in CPT2 activity and subsequent decrease for HCC has been reported by others^44^. The value of AC C16/AC C8 was higher in HBV-associated cirrhosis and HBV-associated HCC than CHB, which presented increased initiation of β-oxidation of long chain FAs as liver disease progresses from CHB to the more advanced stages of cirrhosis and HCC, because the related AcylCoA dehydrogenase is the initial step in β-oxidation of long chain FAs. The ratio of AC C2/carnitine is an estimate of β-oxidation of even-numbered FAs and it seems to be higher in HBV-associated HCC but no significant differences are detected among 3 groups. Overall, based on the above calculated ratios that reflect enzyme activity in the carnitine/β-oxidation pathway, there are perturbations in the handling of long chain FAs for different stages of liver disease. Examination of expression levels for PPAR-α in liver tissue and other factors controlling β-oxidation in liver disease are needed to completely explain these results.

AC C3 reflects the status of the propionyl CoA pool, and propionyl CoA is a byproduct of both isoleucine and valine catabolism^11^. It has been reported that serum albumin and branched-chain amino acids including leucine, isoleucine and valine to tyrosine ratio levels decreased significantly as chronic hepatitis progressed to liver cirrhosis^11^. It is also widely believed that changes in amino acid metabolism play a role in the pathogenesis of many of the complications of cirrhosis, such as encephalopathy^45^, hypoalbuminemia with edema, and IR^46^,^47^,^48^. However, the detailed mechanism of decreased AC C3-OH in cirrhosis remains to be elucidated.

Sphingolipids (SLs) have significant roles in signal transduction^49^, and are involved in the progression of liver disorders, including viral hepatitis, fibrosis, nonalcoholic steatosis hepatitis and HCC^50^,^51^,^52^,^53^,^54^,^55^,^56^. SLs are derived from hydrolysis of SMs by two sphingomyelinases, ie a neutral sphingomyelinase (NSMase) and an acidic sphingomyelinase (ASMase)(Fig. 3c)^57^,^58^. SLs participate in several aspects of the HBV life cycle, and blocking host SL biosynthesis was shown to inhibit HBV replication^56^. Because the liver plays an essential role in the metabolism of SLs, liver diseases are associated with major changes in serum SL concentrations^59^. Hepatic pathology in patients with CHB may be caused by non-HBV-specific inflammation^60^ such as mediation by cytokines that are regulated by SLs^61^,^62^ through NF-κB and MAP kinase pathways^63^. Particularly, SM(18:1/24:0) was found to inversely correlate with MELD score, and positively correlate with inflammation grades in CHB patients^64^; phytosphingosine and dihydrosphingosine were found to be decreased in the sera of HCC patients compared to cirrhosis patients^13^.

Our study showed that 83% of the SMs evaluated were significantly decreased between CHB and later stages of disease progression, HBV-associated cirrhosis and HBV-associated HCC. One particular SM, SM C24:1 was lower in the sera of patients with HBV-associated cirrhosis than in patients with CHB and HBV-associated HCC (Table 2, Fig. 1; Supplementary Fig. S3). While the exact role of SM C24:1 in the progression of HBV-induced liver disease is yet to be fully determined, the significant decrease seen in the HBV-associated cirrhosis stage motivated us to seek a connection with fibrosis. SMs can be metabolized to sphingosine-1-phosphate (S1P) which has been shown to be a potent modulator of fibrosis, exerting pro-fibrotic effects via its ability to increase proliferation and migration activated hepatic stellate cells (HSC). Activated HSCs are the major source of matrix components such as collagen and fibronectin. The proliferative, contractile and fibrinogenic phenotype of activated HSCs has been shown to be due to the activation of their cell-surface S1P_1–3_ receptors by S1P which causes upregulation of α-smooth muscle actin (αSMA), pro-collagen α1(I) and pro-collagen α1(III)^65^,^66^.

There are several limitations in our study. First, a healthy control group was lacking, and the 3 groups of patients were compared to each other, not to a normal baseline. Secondly, although indirect evidence has shown that several lipids were not affected by HBV, it would be better to verify a direct correlation of the observed metabolic alterations with HBV-triggered disease in the presence of an aged-matched and non-viral hepatitis control group. Thirdly, this is a cross sectional study, and cause-and-effect relationship between metabolite markers and disease progression cannot be established well. A follow-up study about the prognosis of patients who have a resolved infection is a necessary topic for future study. Lastly, this study examined only lipid metabolites, and the metabolism of carbohydrates and proteins is likely affected by the HBV-induced liver diseases as well. Further mechanistic research on animals or cells are necessary to investigate alterations in the expression of enzymes and other important factors such as PPARα, PEMT, LPCAT1–4, CPT1,2, LPA, S1P and S1P receptors (Fig. 3). These studies may shed some light on the reason for the altered lipid levels revealed in this study.

In conclusion, clear separations between each two groups were obtained using the PLS-DA scores of 9 lipid metabolites in the circulation, suggesting that the progression of HBV-induced liver diseases have a profound impact on lipid metabolism. Particularly, serum levels of selected long-chain lysoPCs (lysoPC a C18:2, lysoPC a C20:3, lysoPC a C20:4) decreased from CHB to cirrhosis to carcinoma, and concentrations of lysoPC a C20:4 and lysoPC a C20:3 also reflected different levels of disease severity within the group of patients. Overall, serum levels of selected long-chain lysoPCs are promising markers for the progression of HBV-associated liver diseases.

## Methods

### Study population

A total of 136 patients with CHB were recruited, 104 with HBV-associated cirrhosis and 95 with HBV-associated HCC from Longhua hospital affiliated with Shanghai University of Traditional Chinese Medicine(Shanghai, China) between August 2012 and June 2014.

Patients were clinically diagnosed with CHB and HBV-associated cirrhosis according to the “Guideline on prevention and treatment of chronic hepatitis B in China (2010)”^67^. The patient shall be diagnosed with chronic HBV infection if he or she had a previous history of hepatitis B or HBsAg positive for more than 6 months, and presently HBsAg positive and (or) HBVDNA positive. CHB is defined as chronic necroinflammatory disease of the liver caused by persistent HBV infection. It can be subdivided into HBeAg-positive and HBeAg-negative CHB. HBV-associated cirrhosis is developed from CHB, and pathologically defined as diffuse fibrosis accompanied with false lobules. Child-Pugh A Cirrhosis is diagnosed by imaging, biochemical or haematological examinations showing dysfunction of hepatocytes or evidence of portal hypertension (such as hypersplenism with esophageal and gastric varices), or histologically confirmed cirrhosis. Cirrhosis can be clinically classified into two groups, the compensated and the decompensated cirrhosis. Decompensated cirrhosis is usually categorized into Child-Pugh B or C, which includes symptoms with esophageal and gastric variceal bleeding, hepatic encephalopathy, ascites, and/or other severe complications. The diagnosis of cirrhosis in the present study was based on ultrasonography examinations.

HCC was diagnosed according to the “clinical diagnosis and staging criteria of primary hepatic carcinoma”^68^. Clinical diagnosis of HCC can be established if the patient matches the following criteria(1) + (2) a or (1) + (2) b + (3). Specifically, these criteria include (1) evidence with liver cirrhosis and HBV and/or HCV infection (ie, HBV and/or HCV antigen positive); (2) characteristic HCC imaging (CT or MRI) results, (a) if the diameter of the tumor is over 2 cm, (b) if the tumor diameter is 1–2 cm, HCC should be diagnosed by both CT and MRI showing characteristic HCC features; (3) serum AFP level is greater than 400 g/L for 1 months or greater than 200 g/L for 2 months, and such AFP increase is not caused by other factors including pregnancy, reproductive embryonal tumors, active liver disease and secondary liver cancer.

Patients were included if they were diagnosed with CHB, or HBsAg positive and/or HBV-DNA positive cirrhosis and HCC. Patients were excluded if they were younger than 15 or older than 75 years old, pregnant or breast-feeding, co-infected with HCV, hepatitis D virus, human immunodeficiency virus, or if they had diagnosed with severe hepatitis, drug-induced liver disease, alcoholic liver disease and autoimmune liver disease, or if they had received a liver allograft, or had received psychoactive medication. All methods were carried out in accordance with the approved guidelines. All the studies were approved by the Ethics Committee of Shanghai University of Traditional Chinese Medicine. Written informed consent was achieved from all patients.

### Sample collection

Fasting blood samples were collected and centrifuged at 3000 × g for 10 min at room temperature. Sera were aliquoted and stored at −80 °C until use. Demographic characteristics, including age, gender and BMI were recorded during the physical examination. Serum biochemical assays were performed at Longhua hospital using an automatic biochemistry analyzer (Hitachi Ltd, Tokyo, Japan) including ALT, AST, TBIL, DBIL, ALP, GGT, TP, ALB, LDH, TBA, CHE, CREA, TC, TG, GLU, PT, INR, AFP, CEA, RBC, HGB and PLT. Viral load was measured using HBV-DNA detected with the COBAS TaqMan method by Shanghai Adicon Clinical laboratories Inc. MELD Score was calculated according to the equation^69^: MELD Score = 9.6*ln (CREA) (mg/dl) + 3.8*ln (TBIL) (mg/dl) + 11.2*ln (INR) + 6.4*1.

### Sample processing for metabolomics analysis

A targeted metabolomic assay was performed in fasting sera from 335 patients using the Biocrates Absolute IDQ™ p180 kit (BIOCRATES Life Sciences AG, Innsbruck, Austria) following the manufacturer’s instructions. Briefly, serum samples were thawed on ice, then vortexed and centrifuged at 13,000 × g. After 10 μl internal standards (IS) or 10 μl sera were added into the filter wells of a 96-well filter plate, the filter wells were dried by nitrogen, and 50 μl of 5% phenyl-isothiocyanate solution was added into each filter well for derivatization. After incubation, the filter wells were dried, and 300 μl methanol containing 5 mM ammonium acetate were added into each filter well to extract the metabolites. The extract was then centrifuged into the collection wells, and each well was diluted with 300 μl of the running solvent.

The extracted samples were analyzed using a Waters ACQUITY UPLC-TQMS with an electrospray ionization source. The flow injection analysis method was used to quantify 142 small molecule metabolites simultaneously by multiple reaction monitoring. The quantification of metabolites was achieved by reference to appropriate IS. In order to avoid systematic error, samples from different groups were alternately injected.

All data were processed using MassLynx 4.1. software (Waters). The 142 quantified metabolites were: 87 glycerophospholipids which included 73 PC and 14 lysoPC; 40 ACs including AC Cx:y, hydroxylacylcarnitines [AC C(OH)x:y], and dicarboxylacylcarnitines (AC Cx:y-DC); 1 sugar (hexose, sum of six-carbon monosaccharides without distinction of isomers); 14 SMs including SM Cx:y and SM(OH) Cx:y. Metabolites measured with more than 20% missing data (i.e., SM C26:0 and SM C26:1) were excluded from statistical analysis, thus 140 metabolites were quantified in this study (Supplementary Table 1).

### Statistical analysis

Data were expressed as medians (25th, 75th centiles) or frequencies. Statistical comparisons of clinical data within groups were performed using the non-parametric Kruskal-Wallis test for continuous variables, Fisher’s exact test for categorical variables as appropriate using SPSS Statistics 17 (SPSS Inc., Chicago, IL, USA). *P* values were adjusted by the false discovery rate (FDR) method to control the family-wise error rate when performing multiple testing. *P* < 0.05 defined statistical significance. Z-score plot and heat map were generated to visualize variations among groups using R software 3.2.1 (www.r-project.org). Multivariate profile-wide predictive models were constructed using PCA and PLS-DA using SIMCA-P 11.5 (Umetrics, Sweden). The models were validated using stratified 7-fold cross validation. Valid and robust models were assessed by internal cross-validation (R2, Q2Y values) and permutation testing. R2 and Q2 were used to measure “goodness-of-fit” and “goodness-of-prediction” respectively. Values between 0.7 and 1.0, as close to 1, for both R2 and Q2 indicated a very good model with an excellent predictive power^9^. Multiple logistic regression was performed to assess the influence of age, gender and BMI on serum lipid levels.

## Additional Information

**How to cite this article:** Wu, T. *et al*. Serum lipid alterations identified in chronic hepatitis B, hepatitis B virus-associated cirrhosis and carcinoma patients. *Sci. Rep.* **7**, 42710; doi: 10.1038/srep42710 (2017).

**Publisher's note:** Springer Nature remains neutral with regard to jurisdictional claims in published maps and institutional affiliations.

## Supplementary Material

Supplementary Materials

## Figures and Tables

**Table 1:** Demographic information and clinical characteristics of patients.

Variable	CHB	HBV-associated cirrhosis	HBV-associated HCC
Age(ys)	41.00(32.00–52.00)	56.50(48.75–62.25)^***^	56.00(47.00–61.50)^***^
Gender(F/M)	51/85	43/61	14/81^***###^
BMI(kg/m^2^)	23.66(21.96–24.77)	23.44(21.05–25.24)	22.32(19.61–24.16)^***##^
ALT(U/L)	55.50(31.75–109.25)	28.50(19.75–43.25)^***^	40.00(25.00–66.50)
AST(U/L)	44.00(27.00–88.25)	36.00(28.75–54.25)	48.00(32.50–105.50)
TBIL(μmol/L)	14.90(11.47–21.47)	23.75(14.95–38.32)^*^	28.90(14.45–43.80)^**^
DBIL (μmol/L)	5.40(3.50–8.35)	7.10(4.88–14.55)	9.00(4.05–18.35)^*^
ALP(U/L)	66.28(4.50–95.50)	89.00(65.75–108.75)^**^	120.00(83.50–208.00)^***###^
GGT(U/L)	54.50(27.75–111.25)	50.00(26.00–99.25)	110.00(48.00–235.00)^***###^
TP(g/L)	73.45(69.68–76.82)	67.88(62.72–72.80)^***^	69.30(64.20–73.60)^**#^
ALB(g/L)	42.40(39.32–45.32)	36.15(31.85–41.70)^***^	36.10(31.45–40.80)^***^
LDH(U/L)	200.75(160.00–364.25)	187.50(151.12–230.00)^***^	198.00(171.50–249.00)^*^
TBA(μmol/L)	17.95(5.75–24.78)	45.20(18.38–59.62)^***^	37.60(12.45–78.35)^***^
CHE(U/L)	6521.98(4872.50–8114.50)	4066.57(2856.25–5260.25)^***^	3907.00(2526.50–5666.50)^***^
TC(mmol/L)	4.08(3.44–4.51)	3.21(2.84–3.60)^***^	3.70(3.20–4.25)^**###^
TG(mmol/L)	1.20(0.95–1.44)	0.95(0.70–1.11)^***^	1.00(0.70–1.20)^***^
CREA(μmol/L)	58.92(47.65–70.35)	60.80(49.50–71.82)	67.10(55.35–80.40)^***^
GLU(mmol/L)	5.20(4.80–5.63)	5.34(4.94–5.68)	5.29(4.92–5.88)
PT(Sec)	12.50(12.07–13.20)	14.27(13.40–15.62)^***^	14.00(13.10–15.20)^***^
INR	1.09(1.04–1.14)	1.22(1.15–1.31)^***^	1.22(1.12–1.30)^***^
AFP(ng/mL)	5.72(3.33–14.27)	8.62(4.02–23.71)	44.45(6.84–1206.39)^**##^
CEA(ng/mL)	2.10(1.48–2.80)	3.25(1.80–4.05)^***^	2.90(1.80–3.42)^**^
Log10(Viral Load, IU/mL)	4.64(0.00–7.25)	0.00(0.00–3.5)^***^	0.00(0.00–3.18)^***^
RBC(10^12/L)	4.44(4.15–4.74)	3.75(3.31–4.15)^***^	3.90(3.40–4.43)^***^
HGB(g/L)	139.70(132.00–152.00)	118.43(108.00–133.25)^***^	125.43(109.50–141.50)^***#^
PLT(10^9/L)	160.30(123.00–189.50)	69.00(47.00–88.25)^***^	104.00(64.50–168.50)^***###^
MELD score	7.72(7.16–8.55)	10.09(8.96–13.19)^***^	10.81(8.61–13.09)^***^
HBV-DNA Class(Neg/Pos)	48/88	72/32 ^***^	69/26 ^***^
Child-Pugh Class(A/B/C)	—	17/82/5	20/65/10
MELD Score Class (low/high)	115/21	41/63^***^	39/56^***^
Compensated Stage/decompensated Stage	—	60/44	29/66^###^

Values are expressed as medians (25th, 75th centiles) or frequencies. *P* values were calculated from non-parametric Kruskal-Wallis test for continuous variables, Fisher’s exact test for categorical variables for multiple comparisons correction and adjusted by the FDR method. *p < 0.05; **p < 0.01; ***p < 0.001 when compared to CHB. ^#^*p* < 0.05; ^##^*p* < 0.01; ^###^*p* < 0.001 when compared to HBV-associated cirrhosis. HBV-DNA Class, Negative: HBV-DNA < 1E + 03, Positive: HBV-DNA >=1E + 03.

**Table 2:** Metabolites that varied among CHB, HBV-associated cirrhosis and HBV-associated HCC groups.

Metabolites	CHB	HBV-associated cirrhosis	HBV-associated HCC
lysoPC a C18:0 (μmol/L)	34.09(24.77–43.48)	23.68(18.31–35.05)^***^	22.82(15.34–30.84)^***#^
lysoPC a C18:2 (μmol/L)^Δ^	23.66(18.48–29.08)	19.73(13.19–25.76)^**^	15.21(10.54–20.96)^***##^
lysoPC a C20:3 (μmol/L)^Δ^	1.51(1.18–1.94)	1.19(0.84–1.57)^***^	0.97(0.77–1.50)^***#^
lysoPC a C20:4 (μmol/L)^Δ^	4.61(3.51–6.18)	3.52(2.56–4.91)^***^	3.07(2.36–4.34)^***#^
lysoPC a C24:0 (μmol/L)	0.42(0.26–0.55)	0.30(0.19–0.45)^**^	0.28(0.16–0.42)^***#^
PC ae C42:1 (μmol/L)	0.34(0.28–0.40)	0.29(0.21–0.37)^***^	0.26(0.20–0.32)^***#^
AC C18:2(μmol/L)^Δ^	0.09(0.05–0.13)	0.14(0.08–0.19)^***^	0.11(0.07–0.16)^**#^
AC C3-OH (μmol/L)	0.18(0.14–0.22)	0.14(0.12–0.19)^**^	0.17(0.12–0.22)^#^
SM C24:1 (μmol/L)	28.45(18.79–39.37)	21.58(13.02–33.09)^**^	28.02(16.94–48.20)^##^

Values are expressed as medians (25th, 75th centiles). *P* values were calculated from non-parametric Kruskal-Wallis test for continuous variables and adjusted by FDR method. *p < 0.05; **p < 0.01; ***p < 0.001 when compared to CHB. ^#^*p* < 0.05; ^##^*p* < 0.01; ^###^*p* < 0.001 when compared to HBV-associated cirrhosis^. Δ^The metabolites are still independent variables for disease classification considering the effect of age, gender and BMI based on multiple logistic regression. Abbreviations: lysoPC a C18:0, lysophosphatidylcholine acyl C18:0; lysoPC a C18:2, lysophosphatidylcholine acyl C18:2; lysoPC a C20:3, lysophosphatidylcholine acyl C20:3; lysoPC a C20:4, lysophosphatidylcholine acyl C20:4; lysoPC a C24:0, lysophosphatidylcholine acyl C24:0; PC ae C42:1, phosphatidylcholine acyl-alkyl C42:1; AC C18:2, octadecadienylcarnitine; AC C3-OH, hydroxypropionylcarnitine; SM C24:1, sphingomyelin C24:1.

**Table 3:** LysoPC a C20:3 varied with Child-Pugh Class in patients with HBV-associated cirrhosis and HBV-associated HCC.

Variables	HBV-associated cirrhosis			HBV-associated HCC		
	Class A	Class B	Class C	Class A	Class B	Class C
N	17	82	5	20	65	10
Age(ys)	58.00(54.00–61.00)	56.50(48.25–62.75)	53.00(36.00–66.00)	57.00(49.75–63.25)	57.00(47.00–61.00)	51.50(43.25–57.50)
BMI(kg/m^2^)	23.83(22.10–26.03)	23.25(20.62–25.06)	23.78(22.85–25.21)	23.48(21.71–24.80)	22.20(19.38–23.67)	21.46(19.74–24.57)
Gender(F/M)	8/9	33/49	2/3	4/16	9/56	1/9
lysoPC a C18:0 (μmol/L)	23.67(18.87–28.45)	26.31(18.29–35.55)	18.89(6.55–19.50)	25.87(17.09–30.83)	22.77(15.46–31.37)	17.05(10.67–19.84)
lysoPC a C18:2 (μmol/L)^Δ^	22.10(18.75–23.17)	18.76(13.11–26.55)	9.00(8.49–13.00)	17.98(13.07–25.70)	15.19(10.44–20.73)	12.86(7.96–16.66)
lysoPC a C20:3 (μmol/L)^Δ^	1.31(1.01–1.66)	1.19(0.83–1.55)	0.89(0.44–0.94)^*#^	1.25(0.92–1.64)	0.97(0.78–1.47)	0.68(0.54–0.89)^**##^
lysoPC a C20:4 (μmol/L)^Δ^	3.52(2.53–4.05)	3.70(2.70–5.05)	2.17(2.03–2.57)^*###^	3.42(2.53–4.37)	3.21(2.45–4.34)	2.11(1.86–3.06)
lysoPC a C24:0 (μmol/L)	0.33(0.19–0.50)	0.31(0.20–0.45)	0.27(0.21–0.29)	0.35(0.24–0.40)	0.27(0.16–0.42)	0.24(0.10–0.37)
PC ae C42:1 (μmol/L)	0.27(0.21–0.33)	0.30(0.22–0.37)	0.19(0.18–0.28)	0.26(0.20–0.30)	0.27(0.22–0.33)	0.24(0.15–0.29)
AC C18:2 (μmol/L)^Δ^	0.11(0.05–0.14)	0.14(0.09–0.20)	0.16(0.12–0.19)	0.08(0.05–0.11)	0.11(0.08–0.16)^*^	0.12(0.06–0.15)
AC C3-OH (μmol/L)	0.14(0.13–0.18)	0.15(0.12–0.20)	0.12(0.10–0.13)^#^	0.18(0.12–0.23)	0.16(0.12–0.21)	0.16(0.13–0.24)
SM C24:1 (μmol/L)	20.67(15.25–33.84)	21.58(12.16–31.81)	26.94(25.69–36.83)	20.83(14.77–27.00)	32.86(16.48–50.64)^*^	42.22(30.45–64.50)^*^

Values are expressed as medians (25th, 75th centiles) or frequencies. *P* values were calculated from non-parametric Kruskal-Wallis test for continuous variables, Fisher’s exact test for categorical variables for multiple comparisons correction and adjusted by FDR method. *p < 0.05; **p < 0.01; ***p < 0.001 when compared to Class A. ^#^*p* < 0.05; ^##^*p* < 0.01; ^###^*p* < 0.01when compared to Class B. ^Δ^The metabolites are still independent variables for disease classification considering the effect of age, gender and BMI based on multiple logistic regression.

**Table 4:** LysoPC a C20:4 varied with MELD Score and disease progression from CHB to HBV-associated cirrhosis and to HBV-associated HCC.

Variables	CHB		HBV-associated cirrhosis		HBV-associated HCC	
	MELD≤8.99	MELD>8.99	MELD≤8.99	MELD>8.99	MELD≤8.99	MELD>8.99
N	109	27	29	75	30	65
Age(ys)	40.00(32.00–51.00)	45.00(35.50–58.00)	55.00(50.00–62.00)	57.00(48.50–64.50)	56.00(47.00–64.50)	56.00(47.00–61.00)
BMI(kg/m^2^)	23.66(22.03–24.91)	23.67(21.76–24.28)	23.51(20.58–25.71)	23.44(21.16–25.14)	22.07(19.36–23.71)	22.32(19.82–24.22)
Gender(F/M)	45/64	6/21	15/14	28/47	7/23	7/58
lysoPC a C18:0(μmol/L)	35.83(24.65–44.59)	30.38(26.94–40.27)	32.1(21.47–43.82)	22.97(16.04–33.77)^*^	30.66(25.39–36.05)	17.76(14.31–25.86)^***^
lysoPC a C18:2(μmol/L)^Δ^	24.06(19.85–29.05)	20.47(14.09–28.31)	22.46(17.47–29.85)	18.22(12.49–24.97)	20.31(16.81–26.36)	13.25(9.87–19.22)^**^
lysoPC a C20:3(μmol/L)^Δ^	1.51(1.21–1.94)	1.46(1.07–1.94)	1.42(1.09–1.80)	1.10(0.80–1.52)^*^	1.46(0.98–1.68)	0.89(0.72–1.20)^***^
lysoPC a C20:4(μmol/L)^Δ^	4.91(3.61–6.25)	3.92(3.22–5.68)^*^	4.73(3.32–5.88)	3.17(2.34–4.37)^**^	3.86(2.76–5.37)	2.78(2.02–3.76)^**^
lysoPC a C24:0(μmol/L)	0.43(0.26–0.55)	0.35(0.25–0.69)	0.39(0.16–0.54)	0.29(0.20–0.41)	0.24(0.15–0.42)	0.29(0.19–0.41)
PC ae C42:1(μmol/L)	0.34(0.28–0.40)	0.29(0.27–0.35)^*^	0.30(0.22–0.38)	0.29(0.20–0.36)	0.26(0.21–0.30)	0.26(0.20–0.33)
AC C18:2(μmol/L)^Δ^	0.09(0.04–0.13)	0.10(0.07–0.13)	0.13(0.06–0.15)	0.15(0.09-0.20)	0.09(0.07–0.15)	0.11(0.07–0.16)
AC C3-OH(μmol/L)	0.18(0.14–0.22)	0.18(0.14–0.21)	0.18(0.12–0.22)	0.14(0.12–0.18)	0.17(0.14–0.22)	0.16(0.12–0.22)
SM C24:1(μmol/L)	27.16(17.74–38.11)	35.38(23.43–46.77)	21.49(8.14–35.62)	21.67(15.15–31.74)	22.84(14.18–39.60)	32.86(18.55–48.79)

Values are expressed as medians (25th, 75th centiles) or frequencies. *P* values were calculated from non-parametric Kruskal-Wallis test for continuous variables, Fisher’s exact test for categorical variables for multiple comparisons correction and adjusted by the FDR method. *p < 0.05; **p < 0.01; ***p < 0.001 when compared to MELD ≤ 8.99. ^Δ^The metabolites are still independent variables for disease classification considering the effect of age, gender and BMI based on multiple logistic regression.

**Table 5:** 9 metabolites varied in different HBV-DNA in HBV-related diseases.

Variables	HBV-DNA Negative			HBV-DNA Positive		
	CHB	HBV-associated cirrhosis	HBV-associated HCC	CHB	HBV-associated cirrhosis	HBV-associated HCC
N	48	72	69	88	32	26
Age(ys)	45.50(34.00–56.00)	55.00(48.00–63.25)***	55.00(47.00–61.00)***	40.00(30.75–49.00)	58.00(52.75–60.50)***	57.50(48.75–62.00)***
BMI(kg/m^2^)	23.74(22.96–25.18)	23.44(21.41–25.1)	22.60(20.07–24.22)*	23.57(20.96–24.60)	23.34(20.27–25.44)	20.52(18.20–23.36)**#
Gender(F/M)	16/32	32/40	10/59*^###^	35/53	11/21	4/22
lysoPC a C18:0(μmol/L)	35.42(27.53–43.95)	24.90(18.31–34.83)***	22.34(14.99–30.82)***	33.87(24.36–42.51)	23.27(18.35–35.88)*	25.25(17.54–32.28)**
lysoPC a C18:2(μmol/L)^Δ^	25.84(20.64–36.35)	20.14(13.54–26.11)***	15.71(10.48–20.72)***^##^	22.86(17.17–27.23)	17.41(12.71–25.44)*	14.61(10.60–21.41)**
lysoPC a C20:3(μmol/L)^Δ^	1.69(1.22–2.12)	1.17(0.88–1.52)***	0.98(0.77–1.48)***	1.46(1.16–1.81)	1.34(0.76–1.72)	0.96(0.77–1.49)**
lysoPC a C20:4(μmol/L)^Δ^	5.17(3.89–6.75)	3.46(2.56–4.70)***	3.06(2.31–4.27)***	4.42(3.25–5.99)	3.75(2.63–5.01)	3.21(2.57–4.73)**
lysoPC a C24:0(μmol/L)	0.41(0.26–0.55)	0.31(0.20–0.42)*	0.29(0.17–0.40)**	0.44(0.26–0.55)	0.24(0.18–0.54)	0.24(0.16–0.44)*
PC ae C42:1(μmol/L)	0.34(0.29–0.40)	0.29(0.22–0.35)**	0.26(0.20–0.32)***	0.33(0.28–0.40)	0.30(0.20–0.37)	0.26(0.21–0.31)***
AC C18:2(μmol/L)^Δ^	0.08(0.04–0.12)	0.15(0.10–0.21)***	0.11(0.07–0.16)*^##^	0.10(0.06–0.13)	0.11(0.06–0.15)	0.11(0.06–0.16)
AC C3-OH(μmol/L)	0.19(0.16–0.24)	0.14(0.12–0.18)***	0.16(0.12–0.21)**	0.16(0.13–0.20)	0.15(0.10–0.20)	0.18(0.12–0.23)
SM C24:1(μmol/L)	29.81(23.18–38.10)	22.97(15.31–34.02)	27.59(16.48–47.61)	28.02(17.96–40.26)	18.54(10.29–30.55)*	29.33(18.88–49.27)#

Values are expressed as medians (25th, 75th centiles) or frequencies. *P* values were calculated from non-parametric Kruskal-Wallis test for continuous variables, Fisher’s exact test for categorical variables for multiple comparisons correction and adjusted by the FDR method. *p < 0.05; **p < 0.01; ***p < 0.001 when com*p*ared to CHB. ^#^*p* < 0.05; ^##^*p* < 0.01; ^###^*p* < 0.001 when com*p*ared to HBV-associated cirrhosis. ^Δ^The metabolites are still independent variables for disease classification considering the effect of age, gender and BMI based on multiple logistic regression. HBV-DNA Class, Negative: HBV-DNA < 1E + 03, Positive: HBV-DNA >= 1E + 03.
