# Supplementary material for: Serum lipid alterations identified in chronic hepatitis B, hepatitis B virus-associated cirrhosis and carcinoma patients
Source: Sci Rep. 2017 Feb 15;7:42710. doi: 10.1038/srep42710 (PMC5309878; doi:10.1038/srep42710)
Supplement: Supplementary Materials [file srep42710-s1.pdf]

Serum lipid alterations identified in chronic hepatitis B, hepatitis B virus-associated cirrhosis and carcinoma patients

Tao Wu[1,2], Xiaojiao Zheng[3], Ming Yang[2], Aihua Zhao[3], Meng Li[2], Tianlu Chen[3], Jun Panee[4], Wei Jia[3,5,*] , Guang Ji[2,6,*]

[1] Center of Chinese Medical Therapy and Systems Biology, Shanghai University of Traditional Chinese Medicine, Shanghai 201203, China; [2] Institute of Digestive Disease, Longhua Hospital, Shanghai University of Traditional Chinese Medicine, Shanghai 200032, China; [3] Shanghai Key Laboratory of Diabetes Mellitus and Center for Translational Medicine, Shanghai Jiao Tong University Affiliated Sixth People's Hospital, Shanghai 200233, China; [4] Department of Cell and Molecular Biology, John A. Burns School of Medicine, University of Hawaii at Monoa, Honolulu, Hawaii 96813, United States;[5] University of Hawaii Cancer Center, Honolulu, Hawaii 96813, United States; [6] E-institute of Shanghai Municipal Education Committee, Shanghai University of Traditional Chinese Medicine, Shanghai 201203, China.

Correspondence: Wei Jia, PhD, University of Hawaii Cancer Center, Honolulu, Hawaii 96813, United States; or Guang Ji, MD, PhD, Institute of Digestive Disease, Longhua Hospital, Shanghai University of Traditional Chinese Medicine, Shanghai 200032, China. E-mail: wjia@cc.hawaii.edu or jiliver@vip.sina.com.

Contents

**Name**	**Title**
Supplementary Figure 1.	Z-score plots and multivariable model of 140 variables from UPLC-TQMS data among CHB, HBV-associated cirrhosis and HBV-associated HCC groups.
Supplementary Figure2.	PCA and PLS-DA models based on results of plasma UPLC-TQMS profile for each two group comparisons.
Supplementary Figure3.	Heatmap of 9 representative metabolites among CHB, HBV-associated cirrhosis and HBV-associated HCC groups.
Supplementary Table 1.	The metabolomics dataset contains 140 metabolites.
Supplementary Table 2.	*P* values of multiple logistic regression evaluating group differences of 9 metabolites.
Supplementary Table 3.	Correlation between lysoPC a C20:3 and different variables in HBV-associated cirrhosis and HBV-associated HCC.
Supplementary Table 4.	Related ratios in CHB, HBV-associated cirrhosis and HBV-associated HCC groups.

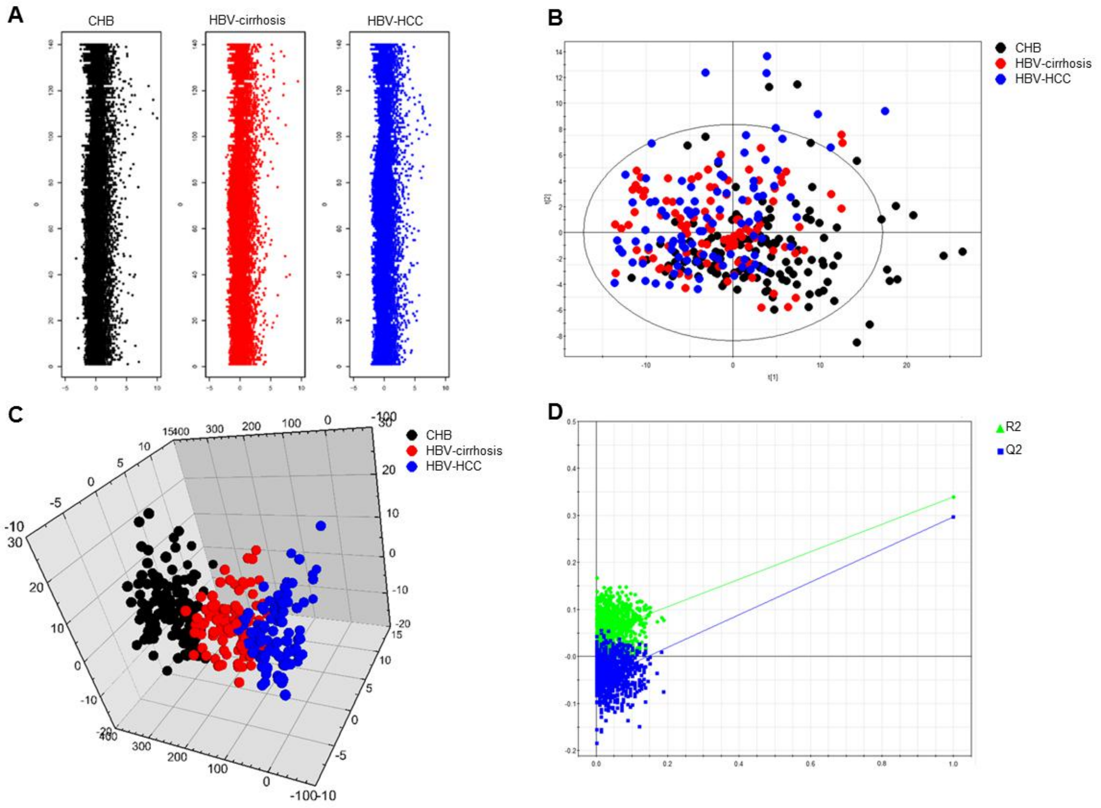

Supplementary Figure1. Z-score plots and multivariable model of 140 variables from UPLC-TQMS data among CHB, HBV-associated cirrhosis and HBV-associated HCC groups.

(**A**) Z-score plots of 140 metabolites in patients with CHB, HBV-associated cirrhosis and HBV-associated HCC. Each point represents one metabolite in one sample, colored by type (black=CHB, red=HBV-associated cirrhosis, blue=HBV-associated HCC). (**B**)Principal components analysis (PCA) scores plot: R2X = 0.653 Q2 = 0.517. (**C**)Partial least squares discriminate analysis (PLS-DA) scores plot: R2X = 0.425 R2Y = 0.182 Q2 = 0.154. (**D**)Permutation analysis.

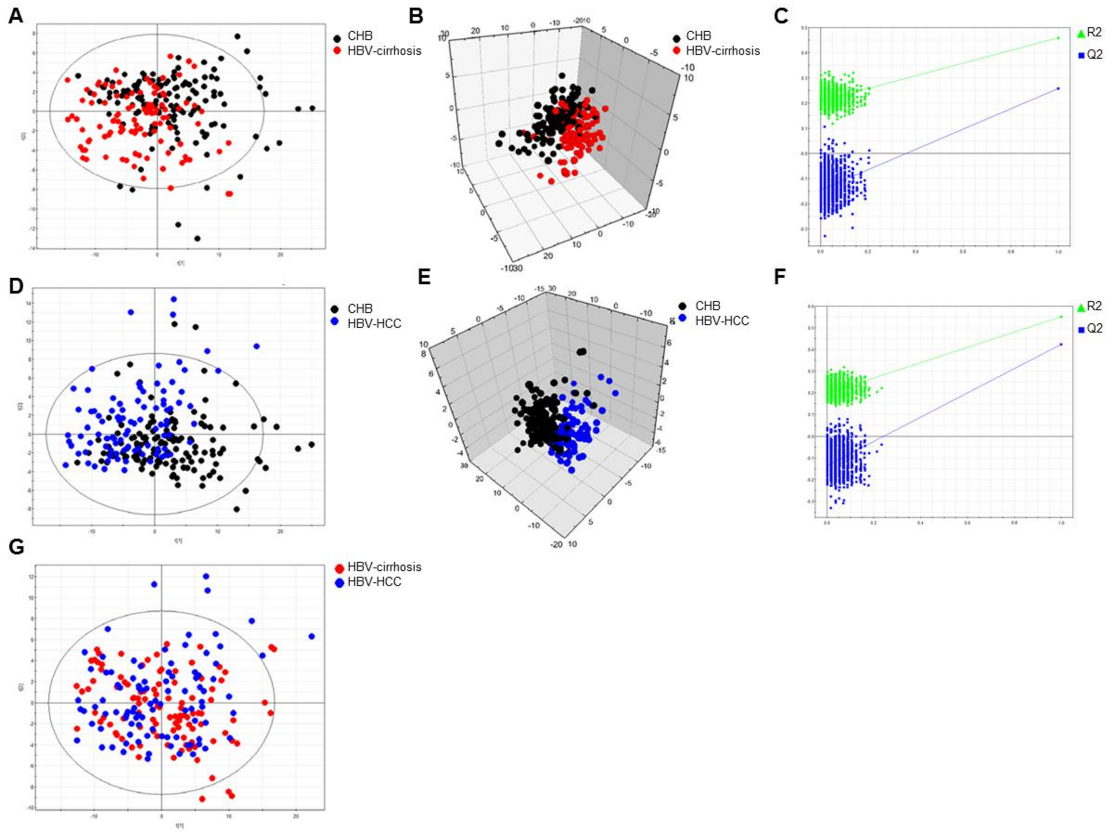

Supplementary Figure 2. PCA and PLS-DA models based on results of serum UPLC-TQMS profile for each two group comparisons.1) CHB versus HBV-associated cirrhosis: (**A**) PCA scores plot: R2X = 0.637 Q2 = 0.494. (**B**) PLS-DA scores plot: R2X = 0.448 R2Y = 0.460 Q2 = 0.257. (**C**) Permutation analysis. 2) CHB versus HBV-associated HCC: (**D**) PCA scores plot: R2X = 0.592 Q2 = 0.475. (**E**) PLS-DA scores plot: R2X = 0.459 R2Y = 0.552 Q2 = 0.424. (**F**) Permutation analysis. 3) HBV-associated cirrhosis versus HBV-associated HCC: (**G**) PCA scores plot: R2X = 0.660 Q2 = 0.493. PLS-DA model could not be established successfully.

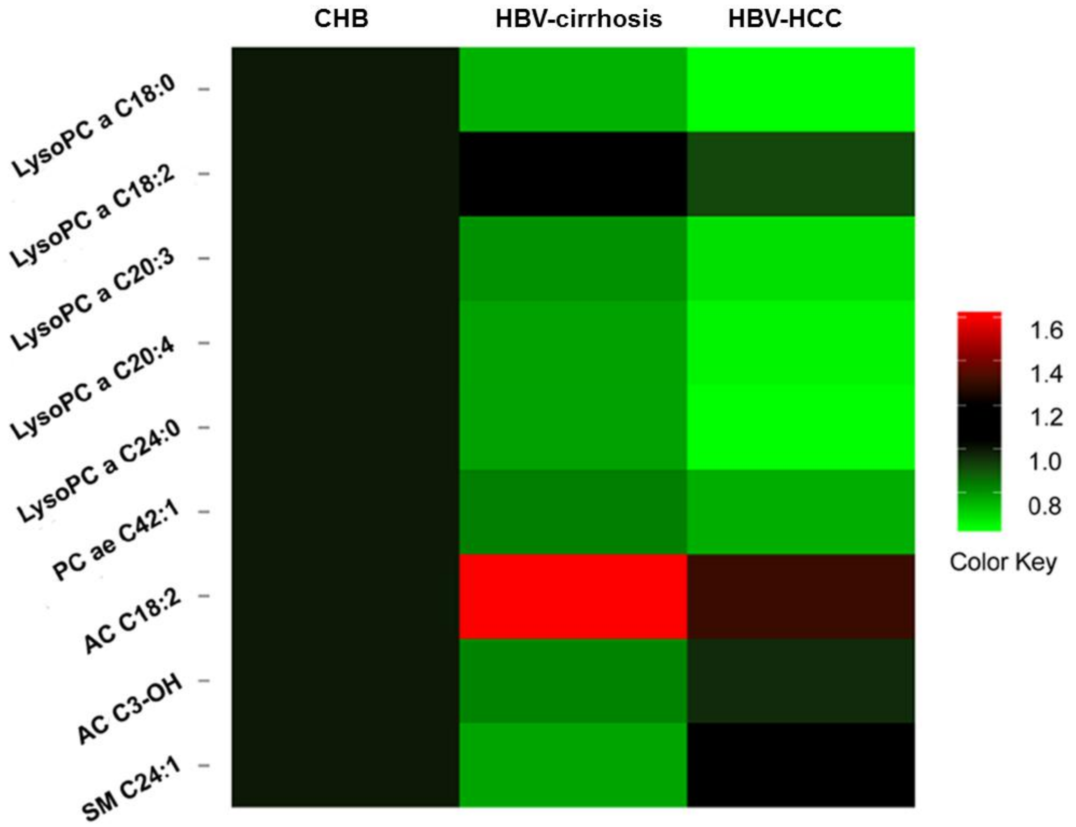

Supplementary Figure 3. Heat map of 9 representative metabolites among CHB, HBV-associated cirrhosis and HBV-associated HCC groups. Heat map shows the Fold Change (FC) of 9 metabolites among three groups compared with CHB. Shade of green and red represent fold decrease and fold increase of a metabolite respectively, in HBV-associated cirrhosis and HBV-associated HCC relative to CHB.

Supplementary Table 1. The metabolomics dataset contains 140 metabolites.

No	Name	Full Name	CHB (N=136)	HBV-associated cirrhosis(N=104)	HBV-associated HCC(N=95)
m001	lysoPC a C14:0	lysoPhosphatidylcholine acyl C14:0	3.08(2.56-3.55)	2.58(2.24-2.94)***	2.52(2.14-2.97)***
m002	lysoPC a C16:0	lysoPhosphatidylcholine acyl C16:0	99.22(77.52-133.29)	76.17(57.96-106.76)**	71.66(49.33-100.02)***
m003	lysoPC a C16:1	lysoPhosphatidylcholine acyl C16:1	2.31(1.7-2.91)	1.88(1.42-2.55)*	1.55(1.18-2.39)***
m004	lysoPC a C17:0	lysoPhosphatidylcholine acyl C17:0	1.27(0.92-1.72)	0.9(0.59-1.38)***	0.79(0.61-1.15)***
m005	lysoPC a C18:0	lysoPhosphatidylcholine acyl C18:0	34.09(24.77-43.48)	23.68(18.31-35.05)***	22.82(15.34-30.84)***#

No	Name	Full Name	CHB (N=136)	HBV-associated cirrhosis(N=104)	HBV-associated HCC(N=95)
m006	lysoPC a C18:1	lysoPhosphatidylcholine acyl C18:1	15.29(11.8-19.88)	13.55(10.72-17.31)	11.41(9.45-16.11)***
m007	lysoPC a C18:2	lysoPhosphatidylcholine acyl C18:2	23.66(18.48-29.08)	19.73(13.19-25.76)**	15.21(10.54-20.96)***##
m008	lysoPC a C20:3	lysoPhosphatidylcholine acyl C20:3	1.51(1.18-1.94)	1.19(0.84-1.57)***	0.97(0.77-1.5)***#
m009	lysoPC a C20:4	lysoPhosphatidylcholine acyl C20:4	4.61(3.51-6.18)	3.52(2.56-4.91)***	3.07(2.36-4.34)***#
m010	lysoPC a C24:0	lysoPhosphatidylcholine acyl C24:0	0.42(0.26-0.55)	0.3(0.19-0.45)**	0.28(0.16-0.42)***#

No	Name	Full Name	CHB (N=136)	HBV-associated cirrhosis(N=104)	HBV-associated HCC(N=95)
m011	lysoPC a C26:0	lysoPhosphatidylcholine acyl C26:0	0.76(0.48-1.05)	0.54(0.27-0.8)***	0.54(0.3-0.8)***
m012	lysoPC a C26:1	lysoPhosphatidylcholine acyl C26:1	0.35(0.23-0.56)	0.26(0.14-0.42)**	0.29(0.13-0.44)**
m013	lysoPC a C28:0	lysoPhosphatidylcholine acyl C28:0	0.74(0.46-1.01)	0.54(0.29-0.78)***	0.47(0.28-0.64)***
m014	lysoPC a C28:1	lysoPhosphatidylcholine acyl C28:1	0.9(0.54-1.15)	0.64(0.34-0.89)***	0.67(0.36-0.84)***
m015	PC aa C24:0	Phosphatidylcholine diacyl C24:0	0.2(0.13-0.26)	0.15(0.1-0.21)***	0.14(0.08-0.19)***
m016	PC aa C26:0	Phosphatidylcholine diacyl C26:0	1.36(1.09-1.65)	1.23(0.8-1.49)*	1.23(0.8-1.51)*
m017	PC aa C28:1	Phosphatidylcholine diacyl C28:1	1.32(1.06-1.63)	1.1(0.83-1.39)***	1.02(0.81-1.28)***

No	Name	Full Name	CHB (N=136)	HBV-associated cirrhosis(N=104)	HBV-associated HCC(N=95)
m018	PC aa C30:0	Phosphatidylcholine diacyl C30:0	2.58(1.92-3.2)	2.29(1.88-3.07)	2.29(1.8-3.08)
m019	PC aa C32:0	Phosphatidylcholine diacyl C32:0	9.34(7.78-12.53)	11.23(8.87-14.09)	11.38(7.62-16.89)*
m020	PC aa C32:1	Phosphatidylcholine diacyl C32:1	7(5.35-10.21)	7.75(5.43-10.25)	7.61(5.18-10.47)
m021	PC aa C32:3	Phosphatidylcholine diacyl C32:3	0.2(0.16-0.24)	0.14(0.11-0.18)***	0.13(0.11-0.17)***
m022	PC aa C34:1	Phosphatidylcholine diacyl C34:1	115.99(94.84-143.03)	100.12(84.83-126.26)	105.85(82.88-134.74)
m023	PC aa C34:2	Phosphatidylcholine diacyl C34:2	308.27(251.76-365.27)	243.2(202.38-318.15)***	245.72(186.75-313.15)*
m024	PC aa C34:3	Phosphatidylcholine diacyl C34:3	8.14(6.2-10.25)	6.68(5.19-9)***	6.72(4.94-8.2)**
m025	PC aa C34:4	Phosphatidylcholine diacyl C34:4	0.7(0.49-0.9)	0.45(0.33-0.63)***	0.42(0.3-0.59)***
m026	PC aa C36:0	Phosphatidylcholine diacyl C36:0	2.36(1.72-3.1)	1.68(1.08-2.17)***	1.47(0.98-1.84)***
m027	PC aa C36:1	Phosphatidylcholine diacyl C36:1	26.16(21.05-34.18)	22.89(18.54-26.87)***	22.08(16.95-29.12)**
m028	PC aa C36:2	Phosphatidylcholine diacyl C36:2	191.77(158.73-227.25)	153.38(119.38-197.96)***	149.19(111.67-184.43)***

No	Name	Full Name	CHB (N=136)	HBV-associated cirrhosis(N=104)	HBV-associated HCC(N=95)
m029	PC aa C36:3	Phosphatidylcholine diacyl C36:3	76.16(61.07-88.22)	65.62(52.85-79.45)***	61.12(49.04-78.12)**
m030	PC aa C36:4	Phosphatidylcholine diacyl C36:4	116.11(90.45-142.88)	83.8(61.16-107.87)***	80.08(61.14-108.13)***
m031	PC aa C36:5	Phosphatidylcholine diacyl C36:5	6.18(4.56-8.94)	4.44(2.91-6.26)***	4.33(3.02-5.69)***
m032	PC aa C36:6	Phosphatidylcholine diacyl C36:6	0.34(0.25-0.46)	0.21(0.14-0.3)***	0.19(0.13-0.28)***
m033	PC aa C38:0	Phosphatidylcholine diacyl C38:0	2.91(2.38-3.69)	2.4(1.79-2.87)***	2.25(1.62-2.87)***
m034	PC aa C38:3	Phosphatidylcholine diacyl C38:3	30.34(23.68-39.06)	24.09(17.31-29.61)***	23.75(18.18-28.41)***
m035	PC aa C38:4	Phosphatidylcholine diacyl C38:4	66.91(53.49-83.73)	46.83(35.41-61.65)***	44.99(33.7-60.33)***
m036	PC aa C38:5	Phosphatidylcholine diacyl C38:5	25.26(19.72-30.85)	18.54(13.4-22.83)***	16.74(12.91-22.66)***
m037	PC aa C38:6	Phosphatidylcholine diacyl C38:6	50.31(37.89-62.51)	30.52(19.03-40.34)***	29.37(21.05-41.42)***
m038	PC aa C40:1	Phosphatidylcholine diacyl C40:1	0.5(0.44-0.58)	0.43(0.37-0.5)***	0.42(0.34-0.49)***
m039	PC aa C40:2	Phosphatidylcholine diacyl C40:2	0.51(0.45-0.64)	0.46(0.38-0.56)*	0.46(0.36-0.6)*

No	Name	Full Name	CHB (N=136)	HBV-associated cirrhosis(N=104)	HBV-associated HCC(N=95)
m040	PC aa C40:3	Phosphatidylcholine diacyl C40:3	0.53(0.44-0.61)	0.48(0.39-0.59)	0.46(0.36-0.62)
m041	PC aa C40:4	Phosphatidylcholine diacyl C40:4	2.78(2.25-3.38)	2.27(1.75-2.71)***	2.22(1.66-2.62)***
m042	PC aa C40:5	Phosphatidylcholine diacyl C40:5	7.04(5.54-8.77)	5.29(3.97-6.54)***	4.81(3.79-6.5)***
m043	PC aa C40:6	Phosphatidylcholine diacyl C40:6	20.77(16.17-28.12)	13.68(9.18-17.8)***	12.6(9.52-17.77)***
m044	PC aa C42:0	Phosphatidylcholine diacyl C42:0	0.62(0.48-0.76)	0.56(0.46-0.7)	0.55(0.44-0.7)
m045	PC aa C42:1	Phosphatidylcholine diacyl C42:1	0.34(0.26-0.42)	0.3(0.22-0.37)**	0.27(0.23-0.36)**
m046	PC aa C42:2	Phosphatidylcholine diacyl C42:2	0.3(0.23-0.35)	0.26(0.2-0.31)***	0.24(0.19-0.32)***
m047	PC aa C42:4	Phosphatidylcholine diacyl C42:4	0.24(0.19-0.28)	0.24(0.18-0.27)	0.22(0.17-0.29)
m048	PC aa C42:5	Phosphatidylcholine diacyl C42:5	0.35(0.27-0.45)	0.29(0.21-0.34)*	0.29(0.22-0.41)*
m049	PC aa C42:6	Phosphatidylcholine diacyl C42:6	0.45(0.37-0.56)	0.38(0.3-0.44)***	0.37(0.32-0.44)***
m050	PC ae C30:0	Phosphatidylcholine acyl-alkyl C	0.31(0.24-0.38)	0.27(0.21-0.36)*	0.26(0.2-0.32)**

No	Name	Full Name	CHB (N=136)	HBV-associated cirrhosis(N=104)	HBV-associated HCC(N=95)
m051	PC ae C30:1	Phosphatidylcholine acyl-alkyl C	0.3(0.22-0.38)	0.26(0.19-0.32)*	0.26(0.2-0.34)*
m052	PC ae C30:2	Phosphatidylcholine acyl-alkyl C	0.12(0.09-0.16)	0.09(0.07-0.12)***	0.1(0.06-0.12)***
m053	PC ae C32:1	Phosphatidylcholine acyl-alkyl C32:1	1.65(1.33-2.13)	1.65(1.43-2.23)	1.65(1.21-2.25)
m054	PC ae C32:2	Phosphatidylcholine acyl-alkyl C32:2	0.38(0.3-0.49)	0.37(0.28-0.45)	0.33(0.26-0.48)*
m055	PC ae C34:0	Phosphatidylcholine acyl-alkyl C34:0	0.74(0.6-0.91)	0.71(0.58-0.84)	0.68(0.52-0.88)
m056	PC ae C34:1	Phosphatidylcholine acyl-alkyl C34:1	4.49(3.5-5.68)	4.63(4.02-5.67)	4.48(3.4-6.57)

No	Name	Full Name	CHB (N=136)	HBV-associated cirrhosis(N=104)	HBV-associated HCC(N=95)
m057	PC ae C34:2	Phosphatidylcholine acyl-alkyl C34:2	6.39(5.35-8.05)	5.88(5.09-7.34)*	5.62(4.16-7.6)**
m058	PC ae C34:3	Phosphatidylcholine acyl-alkyl C34:3	4.36(3.52-5.27)	3.84(2.94-4.92)*	3.4(2.38-4.64)***
m059	PC ae C36:0	Phosphatidylcholine acyl-alkyl C36:0	0.68(0.5-0.9)	0.58(0.46-0.72)**	0.61(0.45-0.84)
m060	PC ae C36:1	Phosphatidylcholine acyl-alkyl C36:1	5.33(4.2-6.49)	4.19(3.22-5.03)***	4.15(3.33-5.2)***
m061	PC ae C36:2	Phosphatidylcholine acyl-alkyl C36:2	6.85(5.81-8.23)	5.53(4.71-7.02)***	5.39(4.45-6.89)***

No	Name	Full Name	CHB (N=136)	HBV-associated cirrhosis(N=104)	HBV-associated HCC(N=95)
m062	PC ae C36:3	Phosphatidylcholine acyl-alkyl C36:3	4.46(3.68-5.54)	4.13(3.36-4.82)*	3.83(2.93-5.06)**
m063	PC ae C36:4	Phosphatidylcholine acyl-alkyl C36:4	10.8(8.69-13.09)	9.23(7.66-11.04)***	8.51(6.62-10.32)***
m064	PC ae C36:5	Phosphatidylcholine acyl-alkyl C36:5	7.44(5.83-9.44)	6.08(4.55-7.11)***	5.43(4.19-6.8)***
m065	PC ae C38:0	Phosphatidylcholine acyl-alkyl C38:0	0.82(0.65-1.05)	0.56(0.38-0.73)***	0.51(0.38-0.68)***
m066	PC ae C38:1	Phosphatidylcholine acyl-alkyl C38:1	2.93(2.29-3.55)	2.29(1.81-2.86)***	2.22(1.75-2.59)***

No	Name	Full Name	CHB (N=136)	HBV-associated cirrhosis(N=104)	HBV-associated HCC(N=95)
m067	PC ae C38:2	Phosphatidylcholine acyl-alkyl C38:2	2.4(2-2.9)	2.06(1.57-2.47)***	1.94(1.56-2.44)***
m068	PC ae C38:3	Phosphatidylcholine acyl-alkyl C38:3	2.85(2.29-3.43)	2.37(1.9-2.86)***	2.32(1.91-2.67)***
m069	PC ae C38:4	Phosphatidylcholine acyl-alkyl C38:4	6.96(5.67-7.95)	5.83(4.69-6.83)***	5.43(4.46-6.41)***
m070	PC ae C38:5	Phosphatidylcholine acyl-alkyl C38:5	11.44(9.25-13.61)	10.14(8.5-12.25)**	10.51(7.5-12.45)**
m071	PC ae C38:6	Phosphatidylcholine acyl-alkyl C38:6	4.92(4-6.14)	3.9(2.88-4.73)***	3.57(2.77-4.32)***

No	Name	Full Name	CHB (N=136)	HBV-associated cirrhosis(N=104)	HBV-associated HCC(N=95)
m072	PC ae C40:1	Phosphatidylcholine acyl-alkyl C40:0	0.94(0.79-1.19)	0.77(0.56-0.96)***	0.67(0.56-0.85)***
m073	PC ae C40:2	Phosphatidylcholine acyl-alkyl C40:2	0.96(0.79-1.18)	0.8(0.64-0.97)***	0.84(0.63-1)***
m074	PC ae C40:3	Phosphatidylcholine acyl-alkyl C40:3	1.06(0.85-1.29)	0.94(0.77-1.13)***	0.89(0.74-1.12)***
m075	PC ae C40:4	Phosphatidylcholine acyl-alkyl C40:4	1.69(1.35-1.98)	1.42(1.19-1.73)***	1.35(1.18-1.58)***
m076	PC ae C40:5	Phosphatidylcholine acyl-alkyl C40:5	2.54(2.05-3)	2.15(1.79-2.55)***	2.05(1.65-2.48)***

No	Name	Full Name	CHB (N=136)	HBV-associated cirrhosis(N=104)	HBV-associated HCC(N=95)
m077	PC ae C40:6	Phosphatidylcholine acyl-alkyl C40:6	3.13(2.55-3.82)	2.54(1.91-2.95)***	2.3(1.86-2.87)***
m078	PC ae C42:0	Phosphatidylcholine acyl-alkyl C42:0	0.77(0.69-0.86)	0.7(0.61-0.76)***	0.67(0.61-0.76)***
m079	PC ae C42:1	Phosphatidylcholine acyl-alkyl C42:1	0.34(0.28-0.4)	0.29(0.21-0.37)***	0.26(0.2-0.32)***#
m080	PC ae C42:2	Phosphatidylcholine acyl-alkyl C42:2	0.36(0.31-0.44)	0.31(0.24-0.36)***	0.29(0.24-0.37)***
m081	PC ae C42:3	Phosphatidylcholine acyl-alkyl C42:3	0.55(0.46-0.67)	0.48(0.41-0.6)**	0.48(0.39-0.6)**

No	Name	Full Name	CHB (N=136)	HBV-associated cirrhosis(N=104)	HBV-associated HCC(N=95)
m082	PC ae C42:4	Phosphatidylcholine acyl-alkyl C42:4	0.73(0.56-0.84)	0.67(0.52-0.82)	0.65(0.53-0.82)
m083	PC ae C42:5	Phosphatidylcholine acyl-alkyl C42:5	1.56(1.28-1.83)	1.4(1.19-1.63)	1.42(1.15-1.8)
m084	PC ae C44:3	Phosphatidylcholine acyl-alkyl C44:3	0.11(0.1-0.14)	0.1(0.08-0.13)**	0.1(0.08-0.12)***
m085	PC ae C44:4	Phosphatidylcholine acyl-alkyl C44:4	0.28(0.23-0.34)	0.26(0.22-0.33)	0.25(0.2-0.32)
m086	PC ae C44:5	Phosphatidylcholine acyl-alkyl C44:5	1.17(0.91-1.56)	1.19(0.97-1.46)	1.2(0.94-1.64)

No	Name	Full Name	CHB (N=136)	HBV-associated cirrhosis(N=104)	HBV-associated HCC(N=95)
m087	PC ae C44:6	Phosphatidylcholine acyl-alkyl C44:6	1.52(1.15-1.81)	1.44(1.15-1.84)	1.46(1.08-1.84)
m088	AC C0	Carnitine	35.02(29.12-42.58)	33.91(27-42.68)	33.48(27.01-46.25)
m089	AC C10	Decanoylcarnitine	0.28(0.21-0.4)	0.21(0.17-0.29)**	0.21(0.17-0.28)***
m090	AC C10:1	Decenoylcarnitine	0.46(0.34-0.57)	0.36(0.29-0.52)*	0.35(0.28-0.46)***
m091	AC C10:2	Decadienylcarnitine	0.06(0.05-0.09)	0.06(0.04-0.07)*	0.05(0.04-0.07)***
m092	AC C12	Dodecanoylcarnitine	0.09(0.07-0.11)	0.07(0.05-0.1)	0.07(0.06-0.1)
m093	AC C12:1	Dodecenoylcarnitine	0.01(0-0.02)	0.01(0-0.03)	0.02(0-0.02)
m094	AC C12-DC	Dodecanedioylcarnitine	0.2(0.13-0.26)	0.18(0.12-0.23)	0.17(0.12-0.22)*
m095	AC C14	Tetradecanoylcarnitine	0.06(0.04-0.09)	0.06(0.04-0.09)	0.06(0.04-0.09)
m096	AC C14:1	Tetradecenoylcarnitine	0.24(0.18-0.29)	0.19(0.15-0.23)***	0.19(0.15-0.24)***

No	Name	Full Name	CHB (N=136)	HBV-associated cirrhosis(N=104)	HBV-associated HCC(N=95)
m097	AC C14:1-OH	Hydroxytetradecenoylcarnitine	0.01(0-0.02)	0(0-0.02)	0.01(0-0.03)
m098	AC C14:2	Tetradecadienylcarnitine	0.06(0.02-0.09)	0.04(0.01-0.09)	0.05(0.02-0.08)
m099	AC C14:2-OH	Hydroxytetradecadienylcarnitine	0(0-0.01)	0(0-0.01)	0(0-0.01)
m100	AC C16	Hexadecanoylcarnitine	0.09(0.06-0.13)	0.1(0.06-0.14)	0.1(0.07-0.14)
m100	AC C16	Hexadecanoylcarnitine	0.09(0.06-0.13)	0.1(0.06-0.14)	0.1(0.07-0.14)
m101	AC C16:1	Hexadecenoylcarnitine	0(0-0)	0(0-0)	0(0-0)
m102	AC C16:1-OH	Hydroxyhexadecenoylcarnitine	0.01(0-0.03)	0.02(0-0.04)	0.02(0-0.04)
m103	AC C16:2	Hexadecadienylcarnitine	0.01(0-0.02)	0.01(0-0.02)	0.01(0-0.02)
m104	AC C16:2-OH	Hydroxyhexadecadienylcarnitine	0(0-0.01)	0(0-0.01)	0(0-0.01)
m105	AC C16-OH	Hydroxyhexadecanoylcarnitine	0(0-0.01)	0(0-0.01)	0(0-0.01)
m106	AC C18	Octadecanoylcarnitine	0.01(0-0.04)	0.01(0-0.04)	0.02(0-0.04)

No	Name	Full Name	CHB (N=136)	HBV-associated cirrhosis(N=104)	HBV-associated HCC(N=95)
m107	AC C18:1	Octadecenoylcarnitine	0.12(0.07-0.17)	0.16(0.11-0.21)**	0.14(0.09-0.19)
m108	AC C18:1-OH	Hydroxyoctadecenoylcarnitine	0(0-0.01)	0(0-0)	0(0-0)
m109	AC C18:2	Octadecadienylcarnitine	0.09(0.05-0.13)	0.14(0.08-0.19)***	0.11(0.07-0.16)**#
m110	AC C2	Acetylcarnitine	5.84(3.88-7.72)	5.49(3.99-7.56)	6.63(4.2-9.36)
m111	AC C3	Propionylcarnitine	0.26(0.19-0.39)	0.26(0.21-0.35)	0.28(0.2-0.39)
m112	AC C3:1	Propenoylcarnitine	0.03(0.02-0.04)	0.03(0.02-0.05)	0.04(0.02-0.05)
m113	AC C3-DC / C4-OH	Hydroxypropionylcarnitine	0(0-0.01)	0(0-0.01)	0(0-0.01)
m114	AC C3-DC-M / C5-OH	Hydroxylcarnitine	0(0-0)	0(0-0)	0(0-0)
m115	AC C3-OH	Hydroxypropionylcarnitine	0.18(0.14-0.22)	0.14(0.12-0.19)**	0.17(0.12-0.22)#
m116	AC C4	Butyrylcarnitine	0.01(0-0.02)	0.01(0-0.01)	0.01(0-0.01)

No	Name	Full Name	CHB<br>(N=136)	HBV-associated<br>cirrhosis(N=104)	HBV-associated<br>HCC(N=95)
m117	AC C4:1	Butenylcarnitine	0.08(0.06-0.1)	0.07(0.05-0.09)	0.07(0.05-0.1)
m118	AC C5	Valerylcarnitine	0.09(0.06-0.13)	0.09(0.06-0.15)	0.08(0.06-0.12)
m119	AC C5:1	Tiglylcarnitine	0.02(0.01-0.02)	0.02(0.01-0.02)	0.01(0.01-0.02)
m120	AC C5:1-DC	Glutaconylcarnitine	0.02(0.01-0.03)	0.02(0.01-0.03)	0.02(0.01-0.03)
m121	AC C5-DC/ C6-OH	Glutarylcarinitine	0.01(0-0.02)	0.01(0-0.02)	0.01(0-0.02)
m122	AC C5-M-DC	Methylglutarylcarnitine	0.01(0-0.23)	0.01(0-0.02)	0.01(0-0.02)*
m123	AC C6 / C4:1-DC	Hexanoylcarnitine	0.02(0.01-0.02)	0.02(0.01-0.02)	0.02(0.01-0.02)
m124	AC C6:1	Hexenoylcarnitine	0.02(0.01-0.02)	0.02(0.01-0.02)	0.02(0.01-0.02)
m125	AC C7-DC	Pimelylcarnitine	0.02(0.01-0.03)	0.02(0.01-0.03)	0.02(0.01-0.03)
m126	AC C8	Octanoylcarnitine	0.23(0.17-0.32)	0.18(0.15-0.25)**	0.19(0.15-0.24)***
m127	AC C9	Nonaylcarnitine	0.03(0.01-0.04)	0.02(0-0.03)*	0.02(0.01-0.03)**

No	Name	Full Name	CHB<br>(N=136)	HBV-associated<br>cirrhosis(N=104)	HBV-associated<br>HCC(N=95)
m128	SM (OH) C14:1	Hydroxysphingomyelin C14:1	3.22(2.63-3.96)	2.71(2.04-3.29)***	2.66(2-3.22)***
m129	SM (OH) C16:1	Hydroxysphingomyelin C16:1	1.86(1.5-2.27)	1.34(1.02-1.72)***	1.37(1.03-1.64)***
m130	SM (OH) C22:1	Hydroxysphingomyelin C22:1	7.88(5.7-11.04)	5.73(3.31-7.92)***	4.71(2.95-6.97)***
m131	SM (OH) C22:2	Hydroxysphingomyelin C22:2	9.6(7.29-13.23)	7.34(4.39-9.99)***	6.77(4.89-9.12)***
m132	SM (OH) C24:1	Hydroxysphingomyelin C24:1	0.01(0-0.11)	0.03(0-0.12)	0.05(0-0.15)
m133	SM C16:0	Sphingomyelin C16:0	89.66(77.98-104.94)	78.52(65.64-93.71)***	73.57(62.72-94.94)***
m134	SM C16:1	Sphingomyelin C16:1	11.88(9.69-13.91)	8.63(7.1-11.73)***	9(6.78-11.07)***
m135	SM C18:0	Sphingomyelin C18:0	18.03(14.45-20.38)	12.12(8.42-14.48)***	11.28(9.13-14.04)***
m136	SM C18:1	Sphingomyelin C18:1	7.61(6.17-8.96)	4.63(3.17-5.98)***	4.54(3.24-5.76)***
m137	SM C20:2	Sphingomyelin C20:2	0(0-0.13)	0.01(0-0.09)	0.03(0-0.11)
m138	SM C24:0	Sphingomyelin C24:0	19.79(16.69-25.42)	15.25(11.98-18.92)***	15.29(11.65-19.02)***

No	Name	Full Name	CHB (N=136)	HBV-associated cirrhosis(N=104)	HBV-associated HCC(N=95)
m139	SM C24:1	Sphingomyelin C24:1	28.45(18.79-39.37)	21.58(13.02-33.09)**	28.02(16.94-48.2)##
m140	Hexose	Hexose	3572.73(2599.63-4207.81)	3277.44(2526.16-4083.57)	3275.3(2416.97-4042.14)

Values are expressed as medians (25th, 75th centiles) (μmol/L). *P* values were calculated from non-parametric Kruskal-Wallis test for continuous variables, Fisher's exact test for categorical variables for multiple comparisons correction and adjusted by the false discovery rate (FDR) method. *, $p<0.05$; **, $p<0.01$; ***, $p<0.001$ when compared to CHB. $^{\#}$, $p<0.05$; $^{\#\#}$, $p<0.01$; $^{\#\#\#}$, $p<0.001$ when compared to HBV-associated cirrhosis. Glycerophospholipids are differentiated with respect to the presence of ester (a) and ether (e) bonds in the glycerol moiety, where two letters (aa = diacyl, ae = acyl-alkyl) denote that two glycerol positions are bound to a fatty acid residue, while a single letter (a = acyl) indicates the presence of a single fatty acid residue. Lipid side chain composition is abbreviated as Cx:y, where x denotes the number of carbons in the side chain and y the number of double bonds.

Supplementary Table 2. *P* values of multiple logistic regression evaluating group differences of 9 metabolites.

Metabolites	CHB vs HBV-associated cirrhosis	CHB vs HBV-associated HCC	HBV-associated cirrhosis vs HBV-associated HCC
lysoPC a C18:0	0.000	0.000	0.059
lysoPC a C18:2$^{\Delta}$	0.007	0.000	0.006
lysoPC a C20:3$^{\Delta}$	0.002	0.000	0.022
lysoPC a C20:4$^{\Delta}$	0.004	0.000	0.013
lysoPC a C24:0	0.007	0.000	0.075
PC ae C42:1	0.000	0.000	0.060
AC C18:2$^{\Delta}$	0.000	0.049	0.017
AC C3-OH	0.000	0.059	0.097
SM C24:1	0.008	0.637	0.008

Multiple logistic regression was performed to access the influence of age, gender and BMI on the performance of representative lipids. $^{\Delta}$ The metabolites are still independent variables for disease classification adjusted for age, gender and BMI(body mass index) based on multiple logistic regression.

Supplementary Table 3. Correlation between lysoPC a C20:3 and different variables in HBV-associated cirrhosis and HBV-associated HCC.

Variables	Corr [a]	Corr [b]
ALT	-0.017	-0.099
AST	-0.113	-0.303**
TBIL	-0.331***	-0.334***
DBIL	-0.318**	-0.354***
ALP	-0.103	-0.291**
GGT	0.13	-0.197
TP	0.19	0.291**
ALB	0.283**	0.399***
LDH	-0.324***	-0.262*
TBA	-0.191	-0.161
CHE	0.258**	0.335***
TC	0.228*	0.182
TG	0.032	0.124
CREA	0.22*	-0.005
GLU	0.126	0.045
PT	-0.194*	-0.297**
INR	-0.209*	-0.236*
AFP	-0.104	-0.16
CEA	-0.112	-0.003

Variables	Corr [a]	Corr [b]
Viral load	0.022	-0.046
RBC	0.22*	0.468***
HGB	0.234*	0.44***
PLT	-0.065	0.064
Child Pugh Scores	-0.21*	-0.373***

Correlation coefficient (Corr) was obtained from Spearman correlation analysis between lysoPC a C20:3 and different variables. [a] means in HBV-associated cirrhosis and [b] means in HBV-associated HCC respectively. *, $p<0.05$; **, $p<0.01$; ***, $p<0.001$ represents P values calculating from Spearman correlation analysis.

Supplementary Table 4. Related ratios in CHB, HBV-associated cirrhosis and HBV-associated HCC groups.

**Ratio**	**Enzymes**	**CHB (N=136)**	**HBV-associated cirrhosis (N=104)**	**HBV-associated HCC (N=95)**
(AC C16+ AC C18)/carnitine	Carnitine palmitoyltransferase 1	0.003(0.002-0.004)	0.003(0.002-0.005)	0.004(0.003-0.004)
(AC C16+ AC C18:1)/ AC C2	Carnitine palmitoyltransferase 2	0.037(0.025-0.049)	0.043(0.031-0.056)*	0.036(0.025-0.050)#
AC C16/AC C8	long chain AcylCoA dehydrogenase	0.406(0.211-0.524)	0.516(0.291-0.700)**	0.529(0.333-0.712)***
AC C2/Carnitine	β-oxidation of even-numbered FAs	0.159(0.129-0.218)	0.158(0.124-0.228)	0.184(0.130-0.254)

Values are expressed as medians (25th, 75th centiles). *P* values were calculated from non-parametric Kruskal-Wallis test. *, p<0.05; **, p<0.01; ***, p<0.001 when compared to CHB. $^{\#}$, p<0.05; $^{\#\#}$, p<0.01; $^{\#\#\#}$, p<0.001 when compared to HBV-associated cirrhosis.
